# Benzo[*a*]pyrene and phenanthrene hemoglobin adducts as biomarkers of longer-term air pollution exposure†

**DOI:** 10.1039/d4em00551a

**Published:** 2024-11-27

**Authors:** Xiangtian Wang, Yihui Ge, Yan Lin, Emily A. Craig, Ruoxue Chen, Richard K. Miller, Emily S. Barrett, Sally W. Thurston, Thomas G. O'Connor, David Q. Rich, Junfeng (Jim) Zhang

**Affiliations:** a Nicholas School of the Environment, Duke University 308 Research Drive, LSRC Room A309 Durham NC 27708 NC USA junfeng.zhang@duke.edu yan.lin@duke.edu +1 (919) 681 7782; b Duke Global Health Institute, Duke University Durham NC USA; c Department of Obstetrics and Gynecology, University of Rochester School of Medicine and Dentistry Rochester NY USA; d Department of Environmental Medicine, University of Rochester School of Medicine and Dentistry Rochester NY USA; e Department of Biostatistics and Epidemiology, Rutgers University School of Public Health Piscataway NY USA; f Department of Public Health Sciences, University of Rochester School of Medicine and Dentistry Rochester NY USA; g Department of Biostatistics and Computational Biology, University of Rochester School of Medicine and Dentistry Rochester NY USA; h Department of Psychiatry, University of Rochester School of Medicine and Dentistry Rochester NY USA; i Department of Neuroscience, University of Rochester School of Medicine and Dentistry Rochester NY USA; j Department of Psychology, University of Rochester Rochester NY USA; k Department of Medicine, University of Rochester School of Medicine and Dentistry Rochester NY USA

## Abstract

Urinary hydroxylated-polycyclic aromatic hydrocarbons (PAHs), with half-life less than 2 days, are established biomarkers of short-term exposure to PAHs, a ubiquitous constituent of air pollution mixture. In this study, we explore the use of PAHs-hemoglobin adducts as biomarkers of longer-term exposure to air pollution by leveraging an extant resource of blood samples collected from 235 pregnant women residing in Rochester, NY. We measured red blood cells for benzo[*a*]pyrene-tetrols (BaPT) and phenanthrene-tetrols (PHET), both of which are hydrolysis products of PAH-hemoglobin adduct. We utilized previously estimated PM_2.5_ and NO_2_ concentrations within the 1 km^2^ grid surrounding each participant's residence, calculated for up to 20 weeks before the blood collection date. Associations between PAHs tetrols and cumulative exposures to ambient PM_2.5_ or NO_2_ over different time periods were examined using a linear mixed-effects model with participant-specific random intercepts adjusting for season, gestation age, maternal age, maternal income level, and pre-pregnancy BMI. We observed positive associations between PHET concentration and cumulative PM_2.5_ exposure over gestational weeks 12–17, and between BaPT concentration and cumulative PM_2.5_ exposure over gestational weeks 3–16 prior to sample collection. Each interquartile range (IQR) increase in 14 week PM_2.5_ exposure (1.26 μg m^−3^) was associated with a 9.02% (95% CI: 0.30%, 17.7%) increase in PHET and a 12.8% (95% CI: 1.09%, 23.5%) increase in BaPT levels. In contrast, no associations were observed between either biomarker and cumulative NO_2_ exposures. These findings underscore the potential of PAH-hemoglobin adducts as longer-term (weeks to 4 months) exposure biomarkers of ambient PM_2.5_.

## Abstract 2

PAH-hemoglobin adduct levels were associated with cumulative PM_2.5_ exposure over the lifespan of red blood cells, demonstrating their utility as biomarkers for longer-term exposure.

## Graphical abstract

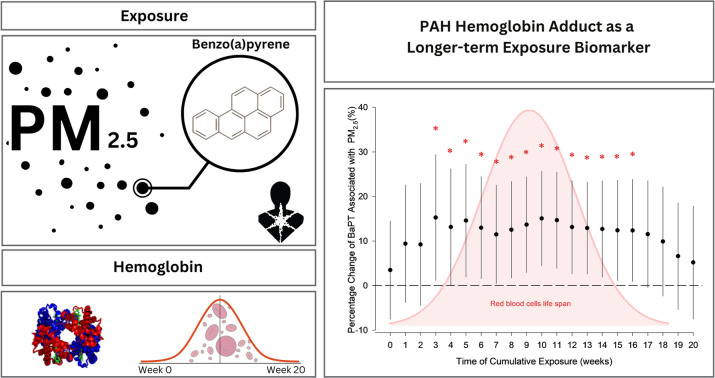

**Environmental significance**

Polycyclic aromatic hydrocarbons (PAHs) are widespread pollutants with significant health effects. Existing biomarkers for PAH exposure, such as urinary hydroxylated PAHs, have a short half-life, limiting their effectiveness for assessing longer-term exposure and chronic health risks. Our study addresses this gap by investigating PAH-hemoglobin adducts as biomarkers for extended exposure to air pollution. We developed a refined method to measure specific PAH-hemoglobin adducts and applied it to a cohort of pregnant women. Our findings highlight the potential of PAH-hemoglobin adducts to serve as reliable indicators of longer-term exposure to pollutants. This approach offers valuable insights into chronic exposure risks and could significantly enhance research on long-term health impacts.

## 1. Introduction

Long-term exposure to air pollution has been associated with the development of chronic health conditions and increased mortality.^1–4^ Currently, assessment of long-term air pollution exposure in epidemiologic studies is mainly based on stationary monitoring and spatial-temporal models that quantify outdoor concentrations only.^5,6^ Feasible methods to assess personal exposures that account for activity patterns in relation to air pollution in different micro-environments are still under development.^7^ Biomonitoring, on the other hand, may capture an individual's exposure by quantifying an internal dose of air pollution exposure. We and others have previously shown in multiple studies that urinary hydroxylated metabolites of polycyclic aromatic hydrocarbons (PAHs) are reliable biomarkers of short-term exposure to air pollution, due to short half-lives (<2 days) of the urinary metabolites.^8–10^ However, biomarkers that can reliably capture exposure over several months are not available.

Benzo[*a*]pyrene (BaP) has often been used as an indicator compound for the PAH mixture due to its relative abundance and toxicity.^11,12^ Previous studies have demonstrated that BaP concentrations correlate with PM_2.5_ and NO_2_ levels in various cities, including Los Angeles and Beijing.^13–15^ BaP undergoes enzymatic activation in the human liver, leading to the formation of BaP diol-epoxides (BPDEs) due to its bay region structure. These BPDEs can bind to hemoglobin (Hb) and persist in the human body for the entire lifespan of Hb, approximately 120 days.^16^ Given their extended lifespan, BaP-hemoglobin adducts have the potential to serve as biomarkers for evaluating exposure over several-month periods. Phenanthrene (PHE), a commonly found three-ring PAH in the environment, shares the same bay region as BaP. Previous studies have identified phenanthrene-tetrols (PHET) in urine and plasma samples.^17,18^ The measurement of PAH-tetrols in red blood cells as hydrolysis products of Hb-PAH adducts, may provide a quantitative method for evaluating longer-term exposure, specifically 3–4 months of exposure, to PAHs or air pollution in general, given that PAHs are ubiquitous constituents of the air pollution mixture.

Here we present the first study (to the best of our knowledge) to explore the use of PAHs-hemoglobin adducts as biomarkers of longer-term air pollution exposure, particularly over a 3 to 4 month period, in residents of a small city of the United States. We used a high-performance liquid chromatography-tandem mass spectrometry (HPLC-MS/MS) to measure benzo[*a*]pyrene-tetrols (BaPT) and PHET in 574 red blood cells collected from 235 pregnant women in Rochester, NY. We examined the associations between both biomarkers and weekly cumulative residential exposure to fine particles (PM_2.5_) and nitrogen dioxide (NO_2_) predicted by high-resolution spatial-temporal models.

## 2. Method

### 2.1 Sample collection

The UPSIDE (Understanding Pregnancy Signals and Infant Development) study was a component of the NIH Environmental Influences on Child Health Outcomes (ECHO) program.^19^ Blood samples were collected from 326 pregnant individuals at each trimester visit between December 2015 and April 2019 in Rochester, a city in western New York. Demographic and socioeconomic data were gathered through questionnaires at enrollment and in each trimester. Exclusion criteria included major endocrine disorders, preterm birth, and obstetric complications threatening pregnancy. The current analysis includes 235 participants whose venous blood samples were collected at least once during pregnancy. Red blood cells, separated after centrifugation for plasma or serum, were stored at −80 °C until analysis. All experiments involving human blood samples were conducted in compliance with the principles of the Declaration of Helsinki. The study protocol was approved by the University of Rochester Research Subjects Review Board and the Duke University Institutional Review Board. Written informed consent was obtained from all participants before sample collection.

### 2.2 PAH-tetrols analytical method

Standard chemicals, r-1,t-2,3,c-4-tetrahydroxy-1,2,3,4-tetrahydrophenanthrene (PHET), were provided by Dr Hecht's laboratory at the University of Minnesota; r-7,t-8,9,c-10-tetrahydroxy-7,8,9,10-tetrahydrobenzo[*a*]pyrene (BaPT-I) was purchased from MRIGlobal (Kansas City, MO, USA); and r-7,t-8,c-9,10-tetrahydroxy-7,8,9,10-tetrahydrobenzo[*a*]pyrene (BaPT-II) was obtained from Toronto Research Chemicals (Ontario, CA).

Red blood cells were processed and analyzed for one metabolite of phenanthrene (PHET) and two metabolites of benzo[*a*]pyrene (BaPT-I and BaPT-II). First, hemolysis was conducted by mixing red blood cells with 0.5 mM EDTA solution in a 4 °C refrigerator for 15 minutes. Second, 10 M NaOH solution was added to the mixture and incubated at 70 Celsius for 60 minutes. Third, after the mixture was cooled down, 10 M HCl was added, and the pH of the mixture was adjusted to 8 using Tris buffer. Fourth, we used ethyl acetate for liquid–liquid extraction twice; and the upper layer was gathered and dried by evaporation under nitrogen gas. Final, the resulting residue was reconstituted in 100 μL of methanol and analyzed using a LC-MS/MS method as below.

The instrument analysis used the atmospheric-pressure chemical ionization (APCI) mode. The gradient program and key instrumental parameters are described in Table S1.† The mass-to-charge ratio is 211.07–165.07 for PHET and 303.3–257.0 for BaPT. The retention times were 5.08 ± 0.15 minutes for PHET, 6.70 ± 0.15 minutes for BaPT-I, and 8.05 ± 0.15 minutes for BaPT-II. The instrumental detection limits, based on of the lowest point the standard curves, were calculated as 12.5 ng mL^−1^ for PHET, 0.02 ng mL^−1^ for BaPT-I, and 0.04 ng mL^−1^ for BaPT-II in pure water. During the pretreatment, we concentrated the samples fourfold. The method detection limits in red blood cell samples were 6.25 ng mL^−1^ for PHET, 0.05 ng mL^−1^ for BaPT-I, and 0.10 ng mL^−1^ for BaPT-II. Among 574 samples analyzed, the detection rates were 83.6% for PHET, 2.60% for BaPT-I, and 57.6% for BaPT-II, respectively. The total concentration of BaP-tetrols (BaPT) was determined by summing the concentrations of BaP-tetrol-I and BaP-tetrol-II. For samples below method detection limits (MDL), we used the area of identifiable peaks to estimate their concentrations when applicable. If no credential peaks were observed, either due to high baseline noises or low signals of the target compound, we used half of the method detection limit (MDL/2).

### 2.3 Assessment of ambient PM_2.5_ and NO_2_ concentrations

Through high-resolution spatiotemporal models previously described to estimate PM_2.5_ concentrations within 1 km^1^ grids of the study area,^20^ and using similar procedures to estimate NO_2_. Participants' daily exposures to PM_2.5_ and NO_2_ were estimated based on the 1 km^2^ grid containing their residence. Daily PM_2.5_ levels were determined by Random Forest models incorporating data from the U.S. Environmental Protection Agency's Air Quality System from 2015 to 2020. These models established connections between contributors to air pollution and measurements. Geocoded addresses were then employed to estimate residential-level PM_2.5_ concentrations based on these models. The same methodology was applied to estimate NO_2_ concentrations.

### 2.4 Statistical analysis

We evaluated exposure–response relationships between PAH-tetrols concentrations and air pollutants exposure data using multivariable linear mixed-effects models with random intercepts at the participant level. In these models, we used weekly cumulative concentrations of PM_2.5_ and NO_2_ to represent continuous exposure. Concentration of PAH-tetrols was a function of cumulative average PM_2.5_ or NO_2_ concentration over a time window preceding the time of blood sample collection (*i.e.*, 1–20 weeks) adjusting for covariates. Exposure to different pollutants (*i.e.*, PM_2.5_ and NO_2_) at different time windows (*i.e.*, 1 week, 1–2 weeks, 1–3 weeks, up to 1–20 weeks) was added to separate models for comparison. We evaluated whether covariates previously associated with both urinary PAH metabolites and any red blood cell biomarkers in our previous studies were confounders.^21,22^ These covariates included maternal age (years), ethnicity (*e.g.*, Hispanic, Non-Hispanic White, African American), education (high school or less, some college, college graduate), income (in categories, *e.g.*, <$15 000, $15 000–$20 000, … >$150 000), smoking status (yes *vs.* no), body mass index (BMI, kg m^−2^), and the season of sample collection (winter, spring, summer, fall). We have used multivariate models to examine the associations of these variables and PAH-tetrols. In addition, we assessed residential proximity to eight potential air pollution sources (*i.e.*, distance to the airport and railroad yard; AADT and truck AADT; and the number of gas stations, crossings, and fast-food restaurants within a 1000 meter buffer). The variables with *p*-value less than 0.05 were kept in the main model.

To ensure the robustness of our findings, several sensitivity analyses were conducted: (a) excluding participants with one or two missing sample collections; (b) including co-pollutants (PM_2.5_ and NO_2_) as covariates in the model; (c) excluding participants who were smoking during pregnancy; (d) excluding participants who moved to another address during pregnancy, (e) using hemoglobin level as a model covariate instead of normalizing PAH-tetrol concentration by it. Statistical significance was based on alpha < 0.05. All analyses were conducted using SAS v.9.4 (© SAS Inc., Cary, N.C.) and R (https://www.r-project.org).

## 3. Results

The participants' mean ± standard deviation age and BMI in early pregnancy were 29.2 ± 4.7 years and 28.8 ± 7.6 kg m^−2^, respectively. A total of 574 blood samples were collected from the 235 participants: 190 (33.1%) in early-pregnancy, 178 (31.0%) in mid-pregnancy, and 206 (35.9%) in late-pregnancy. Among the participants, 143 individuals (60.9%) provided three samples, 53 (26.6%) contributed two samples, and 39 (16.6%) provided only one sample.

Participants' mean ± standard deviation PHET concentrations were 12.9 ± 9.15 ng mL^−1^, while their mean ± standard deviation BaPT concentrations were 0.218 ± 0.161 ng mL^−1^. The hemoglobin concentration was 7.88 ± 2.45 g dL^−1^ (Table 1). Normalized by hemoglobin concentration, mean ± standard deviation PHET concentrations were 167 ± 110 ng g^−1^ Hb. There were no differences in PHET levels between early-, mid-, and late-pregnancy (1st: 175 ± 150 ng g^−1^ Hb, 2nd: 166 ± 93.5 ng g^−1^ Hb; and 3rd: 160 ± 72.4 ng g^−1^ Hb). In contrast, there was an increasing trend in BaPT concentrations from the early- to late-pregnancy (early-pregnancy: 2.27 ± 1.82 ng g^−1^ Hb, mid-pregnancy: 3.06 ± 2.38 ng g^−1^ Hb; and late-pregnancy: 3.57 ± 2.61 ng g^−1^ Hb, *p*-value < 0.001).

**Table 1:** Characteristics of air pollutant exposure and PAH-tetrols biomarker concentrations by trimester^a^

	Trimester 1 (*N* = 190)	Trimester 2 (*N* = 178)	Trimester 3 (*N* = 206)	Overall (*N* = 574)
**PHET (ng mL** ^**−1**^ **)**				
Mean (SD)	14.1 (13.3)	12.2 (6.79)	12.3 (5.43)	12.9 (9.15)
Median [min, max]	11.1 [0.20, 106]	11.6 [0.02, 32.4]	11.3 [0.20, 28.8]	11.3 [0.02, 106]

**BaPT (ng mL** ^**−1**^ **)**				
Mean (SD)	0.177 (0.140)	0.215 (0.148)	0.259 (0.179)	0.218 (0.161)
Median [min, max]	0.129 [0.069, 0.712]	0.186 [0.069, 0.697]	0.232 [0.069, 0.842]	0.173 [0.069, 0.842]

**Hemoglobin (g dL** ^−1^ **)**				
Mean (SD)	8.04 (2.06)	7.82 (2.95)	7.79 (2.31)	7.88 (2.45)
Median [min, max]	7.77 [3.27, 15.1]	7.34 [1.95, 25.1]	7.42 [3.22, 15.4]	7.55 [1.95, 25.1]

**PM**_**2.5**_				
Mean (SD)	6.93 (1.76)	6.76 (2.04)	7.00 (2.04)	6.90 (1.95)
Median [min, max]	6.60 [3.79, 12.6]	6.18 [3.75, 14.0]	6.84 [3.14, 14.2]	6.52 [3.14, 14.2]

**NO**_**2**_				
Mean (SD)	11.4 (5.31)	10.3 (5.02)	9.94 (5.29)	10.5 (5.24)
Median [min, max]	11.2 [1.73, 24.3]	10.6 [1.36, 22.6]	10.6 [0.600, 24.9]	10.8 [0.600, 24.9]

a PHET – concentration of phenanthrene tetrol. BaPT – concentration of benzo(*a*)pyrene tetrols. PM_2.5_ – concentration of PM_2.5_ on the day RBC samples collected. NO_2_ – concentration of NO_2_ on the day RBC samples collected.

Mean ± standard deviation cumulative exposures to PM_2.5_ and NO_2_ over the 20 weeks preceding sample collection were 6.78 ± 1.12 μg m^−3^ and 10.6 ± 4.98 ppb, respectively. There were no significant differences in PM_2.5_ and NO_2_ concentrations from early-, mid-, and late-pregnancy blood collection dates. PM_2.5_ concentrations were 6.90 ± 1.95 μg m^−3^ overall, with values of 6.76 ± 2.04 μg m^−3^ in early-pregnancy, 6.93 ± 1.76 μg m^−3^ in mid-pregnancy, and 7.00 ± 2.04 μg m^−3^ in late-pregnancy. NO_2_ concentrations were 10.5 ± 5.2 ppb overall, with values of 10.3 ± 5.0 ppb in early pregnancy, 11.4 ± 5.3 ppb in mid-pregnancy, and 9.9 ± 5.3 ppb in late pregnancy.

**Fig. 1:**
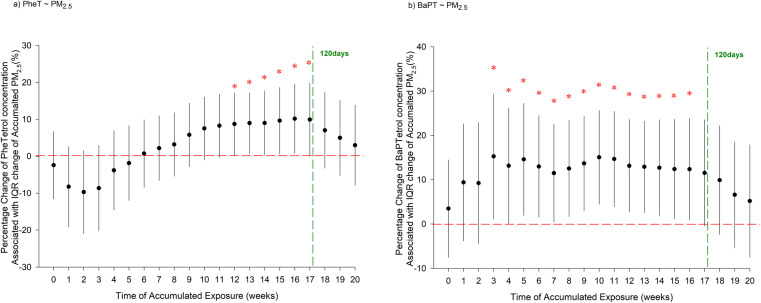
Changes of PAH-tetrols concentration with an IQR change of weekly cumulative PM_2.5_ exposure (a) phenanthrene-tetrols (PHET) adjusted by hemoglobin levels; (b) benzo(*a*)pyrene-tetrols (BaPT) adjusted by hemoglobin levels. Generated using mixed-effects models with random intercepts of study participants and controlling for age, gestation age, pre-pregnancy body mass index, income groups, education. * Indicates *p* < 0.05.

In multivariate models, we found that season, gestational age, pre-pregnancy BMI, education level, and income level may influence adduct concentrations (Fig. S1†). After controlling for these covariates, increasing cumulative PM_2.5_ concentrations over the 12–17 weeks before the blood sample was collected were associated with increases in PHET levels (Fig. 1). Similarly, increasing BaPT levels were associated with increasing cumulative PM_2.5_ concentrations over the 3–16 weeks before sample collection. Specifically, each IQR increase in 14 week PM_2.5_ concentration (*i.e.*, 1.63 μg m^−3^) was associated with an 8.71% (95% CI: 0.30%, 17.1%) increase in PHET concentration and a 12.4% (95% CI: 1.14%, 23.6%) increase in BaPT concentration. Neither PHET nor BaPT showed significant associations with air pollution sources (Table S3†). In different sensitivity analyses, there were no noticeable changes in the associations between PM_2.5_ and PHET or BaPT, confirming the robustness of the main analysis results (Fig. S2†). No associations of either biomarker with NO_2_ exposure were observed (Fig. S3†).

## 4. Discussion

This study was the first to establish a link between PAH-hemoglobin adducts (BaP and PHE) and exposure to PM_2.5_ over a period of up to 4 months. Our findings provided evidence that air pollution exposure, even at low levels, contributed to increased levels of carcinogenic PAHs in the blood of pregnant women. These results also support the promising use of PAH-hemoglobin adducts as exposure biomarkers of longer-term exposure to ambient and indoor PM_2.5_. This study leveraged the resource from a cohort of pregnant women. There is no reason to expect that the findings were specific to pregnant women.

Numerous studies, including ours, have shown a correlation between hydroxylated metabolites of PAHs in urine with short-term PM_2.5_ exposure.^13,20,23,24^ Some studies also reported the detection of BaPT and PHET in urines.^17,25^ However, these investigations have primarily concentrated on short-term exposure due to the short biological half-life of urinary PAH metabolites. In contrast, the mean lifespan of red blood cells (RBCs) ranges around 120 days, with variations between 70 and 140 days.^26,27^ Although this study cannot directly reveal information on the biological half-life of PAH-hemoglobin adducts, we found a notable increase in PAH-hemoglobin adducts in association with cumulated exposure to PM_2.5_ over 84–119 days (for PHET) or 21–112 days (for BaPT) prior to the sample collection. These time windows are aligned with the lifespan of hemoglobin (70–140 days).

The concentrations of BaP-hemoglobin adduct in our study were comparable to those previously reported. The mean Hb-BaP adduct level of 42 non-smokers was 0.068 fmol mg^−1^ Hb and 0.126 fmol mg^−1^ Hb among 6 nonsmokers, respectively.^27,28^ In a normal pregnancy, the increase in plasma volume exceeds the rise in red blood cell mass, resulting in a reduction in hemoglobin concentration.^29^ In our study, we observed a decrease in hemoglobin levels during pregnancy. After adjusting for hemoglobin concentration, the mean ± standard deviation of BaP adduct levels was 0.093 ± 0.071 fmol mg^−1^ Hb, consistent with previous findings in non-pregnant populations.

To the best of our knowledge, there has been no published data on concentrations of PHET in red blood cells. We found that the average PHET concentration was 58.9 times higher than that BaPT in red blood cells, which is in the same range as the fold difference between PHE and BaP in ambient air. For example, the median annual concentration ratio between ambient PHE and BaP was 40.0 in Proto from 2004 to 2014 and 74.5 in Hong Kong from 1998 to 2016.^30,31^ This indicates that the relative abundance of PHET and BaPT in red blood cells were consistent with the relative abundance of PHE and BaP in the ambient air.

BaP-hemoglobin adducts have been recognized and studied for many years mainly for the purpose of studying toxicity mechanisms.^32,33^ It is well-recognized that the metabolic activation of PAHs is crucial to their carcinogenic effects. Thus, measuring diols with different toxicities offers new opportunities to use metabolite ratios to track changes in PAH metabolic activation.^34^ The epoxide at the 9,10 positions in the bay region of BaP is more carcinogenic *in vivo* than the other (Fig. S4†). After hydrolysis, there are two enantiomeric products of each BaP epoxide. Previous studies have only observed the two BaPT of these four enantiomers in serum or plasma.^27,32^ Our method can separate and measure these two observed BaPT, allowing for a detailed analysis of BaP metabolism. This provides crucial insights into the metabolic pathways and their implications for health. Although in the current study, we cannot calculate the ratio of BaPT due to the low detection rate of BaP-I, it is likely that the BaP-I can be more frequently detected in populations with high exposures. This warrants further investigations in individuals occupationally or environmentally exposed to higher levels of BaP (*e.g.*, people working or living close to coal and biomass combustion sources, or smoker).^28,35,36^

Previous studies have shown that PAH metabolites were linked to ambient air pollutants, such as urinary amino-PAHs associated with short-term (1–2 days) exposure to NO_2_.^37,38^ In this study, we present PAH metabolites in blood as long-term biomarkers that capture cumulative exposure from multiple sources, including ambient air, indoor air, diet, and smoking (or secondhand smoke). While various sources, such as secondhand smoke and grilled food consumption, could contribute to blood BaP levels and may include occasional (non-steady) exposures,^33,39^ our study found that ambient PM_2.5_ had a significant and measurable impact on BaP-hemoglobin adduct concentrations. Therefore, regarding long-term exposure, occasional exposure may be less impactful than continuous exposure, even at low-level.

In this same cohort of pregnant women, we found that urinary 1-hydroxylpyrene (1-OHP) was strongly related to PM_2.5_ exposure but not to NO_2_,^21^ consistent with findings for PAH-tetrols in red blood cells. Furthermore, 1-OHP has been correlated with traffic-related sources, including proximity to airports, railway yards, and AADT. However, in our analysis, PHET and BaPT did not show significant associations with these traffic sources. This difference suggests that PAH metabolites may follow different exposure pathways, with dietary sources playing a larger role in long-term PAH biomarker accumulation compared to traffic-related air pollution. For short-term exposure biomarkers, a spot urine was less likely to capture an acute dietary source of PAHs (*e.g.*, eating grilled food in the past 1 to 2 days) than a blood sample to capture such as source in the past months. These findings highlight the need for further research into long-term biomarkers of environmental pollutants and the role of dietary exposure in PAH accumulation.

We calculated Spearman correlation coefficients to evaluate the associations between biomarkers (BaPT and PHET) and cumulative PM_2.5_ concentrations over time. The correlations for PheT with cumulative PM_2.5_ peaked between weeks 9 and 13, with coefficients ranging from 0.08 to 0.18, while BaPT exhibited weaker correlations, ranging from 0.02 to 0.10, mainly concentrated in weeks 1 to 10 (Fig. S5†). These correlations, consistent with the association analysis indicate that BaP and PHE tetrol are linked to longer-term exposure. However, the low correlation coefficients (<0.2) suggest that ambient PM_2.5_ concentrations are not strong predictors of biomarker levels unless adjusted for other influencing factors.

In the association analysis, PHET shows a significant positive association with cumulative PM_2.5_ from weeks 13 to 16, while BaPT is significantly associated from weeks 3 to 16. This timing suggests a 4 week shift between the peak periods identified by correlation coefficients and those observed in the mixed-effects model, which be attributed to factors such as gestational stage and seasonal effects, both of which can influence PAH exposure and biomarker formation. For example, seasonal patterns in northern cities, particularly winter heating-related emissions, increase PM_2.5_ and PAH levels, which may elevate biomarker concentrations.^40^ These seasonal pollution patterns, combined with gestational physiological changes, could influence the timing of biomarker responses, potentially contributing to the observed 4 week offset.

It should be noted that our study was conducted in a city with low air pollution levels among pregnant women. Besides air pollution, dietary exposure and the gestational physiological changes of xenobiotic metabolism and red blood cell biology may also have substantial influence on biomarker levels. Despite the weak correlation, we provided first evidence to support the use of BaP and PHE tetrol as potential longer-term exposure to air pollution exposure. In settings where people's PAH exposure is dominated by combustion-generated air pollution, we would expect that BaPT and PHET had a stronger predicting power for long-term exposure to air pollution.

Our findings suggest that PAH-hemoglobin adducts can serve as biomarkers for long-term exposure to ambient PM_2.5_ under specific conditions: (1) ambient PAH concentrations must be highly correlated with PM2.5 levels (*i.e.*, combustion sources are a significant contributor to ambient PM_2.5_). (2) Other PAH exposure sources (*e.g.*, indoor combustion, dietary intake) should either be negligible or remain constant for up to 20 weeks, as captured by the long-term biomarkers. These conditions appear to have been met by the participants in this study—pregnant women residing in Rochester, NY.

The detection rate of BaPT in our study was relatively low, and a large fraction of the data were based on chromatographic peaks under MDL. Hence, the uncertainty for these below MDL values was high, which may decrease statistical power. Nevertheless, with a relatively large sample size, we were still able to detect positive associations between PM_2.5_ exposure and sum of the two isomers.

## 5. Conclusion

This study demonstrates the potential of PAH-hemoglobin adducts, specifically BaPT and PHET, as biomarkers of longer-term exposure to air pollution. By leveraging blood samples from pregnant women and utilizing cumulative estimates of ambient PM_2.5_ and NO_2_, we observed significant associations between PAH adduct levels and PM_2.5_ exposure over extended time periods, particularly during critical weeks of gestation. In contrast, no associations were found with NO_2_ exposure. These findings suggest that PAH-hemoglobin adducts may serve as valuable biomarkers for assessing long-term ambient PM_2.5_ exposure, complementing urinary hydroxylated-PAHs, which are established markers of short-term exposure. This research highlights the utility of PAH-adducts in capturing longer-term environmental health risks.

## Data availability

Data collected from human participants is not available for public access because they contain information that could compromise the privacy of research participants. However, the de-identified datasets generated and/or analyzed during the current study are available from the authors for external validation.

## Author contribution

Xiangtian Wang: conceptualization, laboratory analysis, data analysis, writing—original draft preparation. Yihui Ge: laboratory analysis, data analysis. Yan Lin: conceptualization, laboratory analysis, data analysis, writing—reviewing and editing. Emily A. Craig: laboratory analysis. Ruoxue Chen: laboratory analysis. Richard K. Miller: subject recruitment and sample collection, writing—reviewing and editing. Emily S. Barrett: subject recruitment and sample collection, writing—reviewing and editing. Sally W. Thurston: data analysis, writing—reviewing and editing. Thomas G. O'Connor: subject recruitment and sample collection, writing—reviewing and editing. David Q. Rich: supervision, fund acquisitions, writing—reviewing and editing. Junfeng (Jim) Zhang: conceptualization, supervision, fund acquisitions, writing—reviewing and editing.

## Conflicts of interest

There are no conflicts to declare.

## Supplementary Material

EM-027-D4EM00551A-s001
