# Supplementary material for: Benzo[a]pyrene and phenanthrene hemoglobin adducts as biomarkers of longer-term air pollution exposure
Source: Environ Sci Process Impacts. 2024 Nov 27;27(1):146–53. doi: 10.1039/d4em00551a (PMC11606450; doi:10.1039/d4em00551a)
Supplement: EM-027-D4EM00551A-s001 [file EM-027-D4EM00551A-s001.pdf]

Supplementary Information (SI) for Environmental Science: Processes & Impacts.
This journal is © The Royal Society of Chemistry 2024

Supporting Information

Benzo[a]pyrene and Phenanthrene Hemoglobin Adducts as Biomarkers of Longer-Term Air Pollution Exposure

Xiangtian Wang,[1#] Yihui Ge,[1#] Yan Lin,[1,2*] Emily Craig,[1] Ruoxue Chen,[1] Richard K. Miller,[3,4] Emily S. Barrett,[3,5,6] Sally W. Thurston,[4,7] Thomas G. O'Connor,[3,8,9,10,] David Q. Rich,[4,6,11] and Junfeng (Jim) Zhang[1,2*]

1. Nicholas School of the Environment, Duke University, Durham, NC, USA
2. Duke Global Health Institute, Duke University, Durham, NC, USA
3. Department of Obstetrics and Gynecology, University of Rochester School of Medicine and Dentistry, Rochester, NY, USA
4. Department of Environmental Medicine, University of Rochester School of Medicine and Dentistry, Rochester, NY, USA
5. Department of Biostatistics and Epidemiology, Rutgers University School of Public Health, Piscataway, NY, USA
6. Department of Public Health Sciences, University of Rochester School of Medicine and Dentistry, Rochester, NY, USA
7. Department of Biostatistics and Computational Biology, University of Rochester School of Medicine and Dentistry, Rochester, NY, USA
8. Department of Psychiatry, University of Rochester School of Medicine and Dentistry, Rochester, NY, USA

[9.] Department of Neuroscience, University of Rochester School of Medicine and Dentistry, Rochester, NY, USA

[10.] Department of Psychology, University of Rochester, Rochester, NY, USA

[11.] Department of Medicine, University of Rochester School of Medicine and Dentistry, Rochester, NY, USA

# These authors contribute equally to this work.

* Corresponding Author: Yan Lin, PhD and Junfeng (Jim) Zhang, PhD

Yan Lin, PhD

Nicholas School of the Environment & Duke Global Health Institute, Duke University, 308 Research Drive, LSRC Room A309, Durham, NC 27708

Email: yan.lin@duke.edu

Junfeng (Jim) Zhang, PhD

Nicholas School of the Environment & Duke Global Health Institute, Duke University, 308 Research Drive, LSRC Room A309, Durham, NC 27708

Phone number: (919) 681-7782

Email: junfeng.zhang@duke.edu

List of Figure and Table in Support Information

Figure S1. Fold difference in PAH-tetrols (PHET and BaPT) levels in association with age, race/ethnicity, education, household income, pre-pregnancy BMI, smoker, gestational age, and season of sample collection.

Figure S2. Sensitive analysis

Figure S3. The Change of PAH-Tetrols Concentration with an IQR Change of Weekly Cumulative NO_2 Exposure

Figure S4. Metabolic Activation of Benzo(a)pyrene: Formation of Diols, Di-Epoxides, and Tetrols.

Table S1-1. HPLC mobile phase gradient program

Table S1-2. Parameters used for the HPLC-MS/MS analysis

Table S2. Estimated percentage change of PAH-tetrols concentration associated with each interquartile range (IQR) change in cumulative $PM_{2.5}$ weekly concentrations.

Table S3. Percentage changes of PAH-tetrols concentrations with one interquartile range (IQR) increase in proximity to a source traffic variable

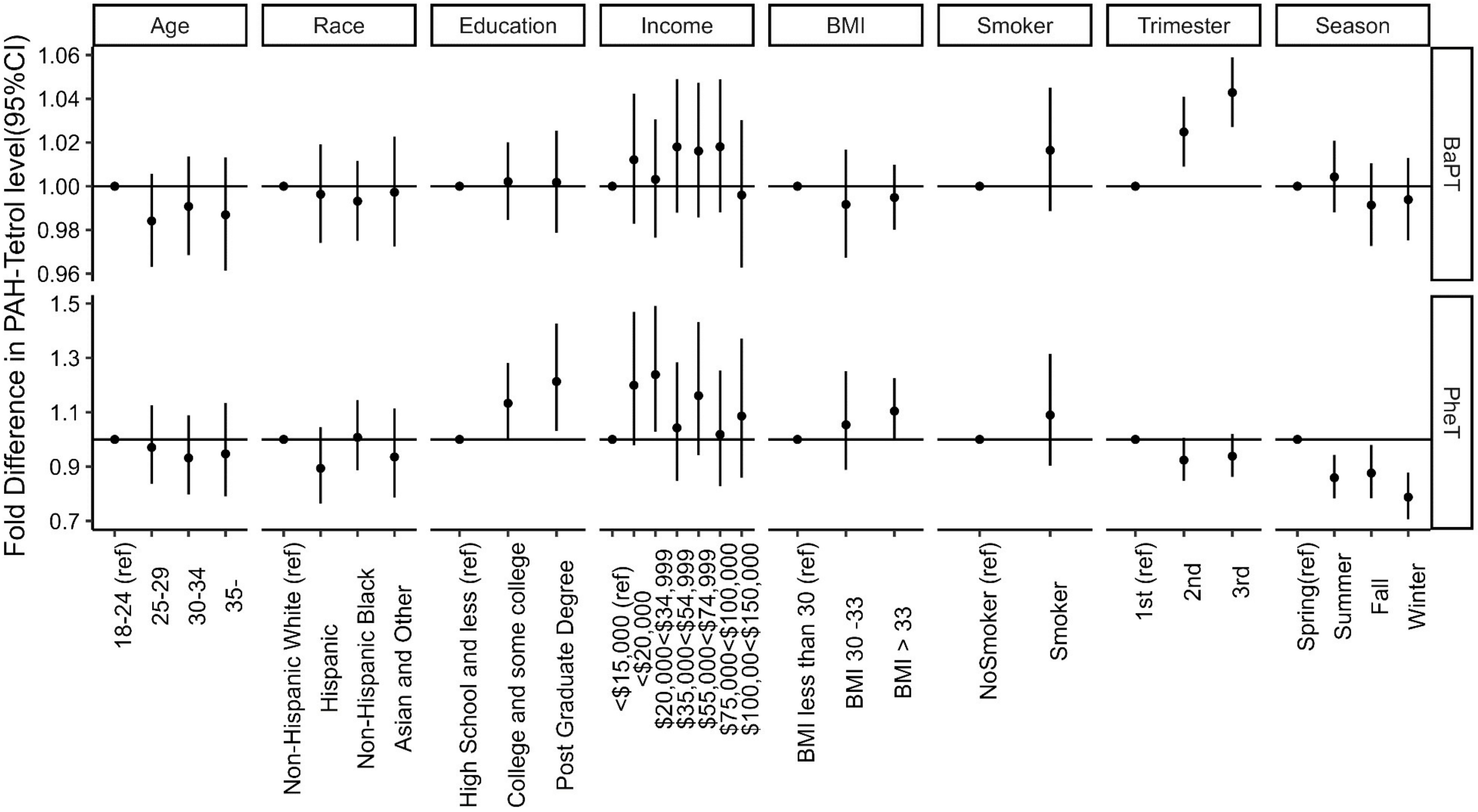

Figure S1. **Fold difference in PAH-tetrols (PHET and BaPT) levels in association with age, race/ethnicity, education, household income, pre-pregnancy BMI, smoker, gestational age, and season of sample collection.** Differences were tested by linear mixed-effects models with random intercepts of study participants.

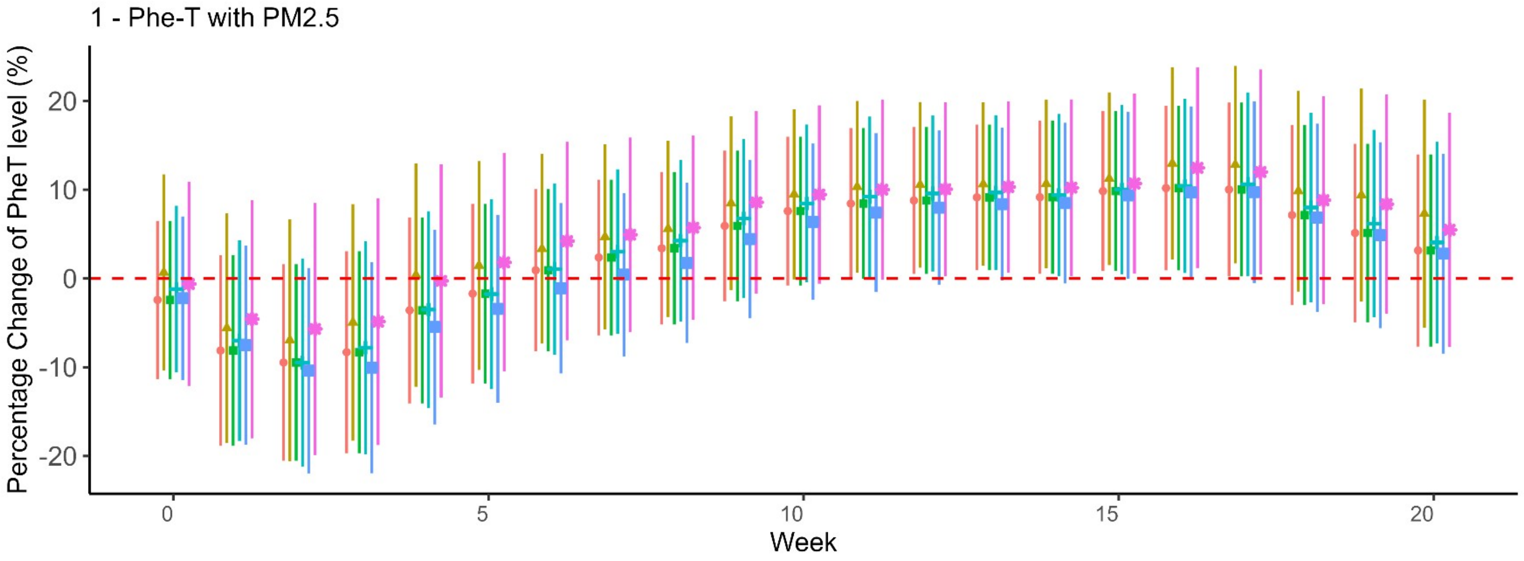
1 - Phe-T with PM2.5
Percentage Change of PheT level (%)
20
10
0
-10
-20
0
5
10
15
20
Week

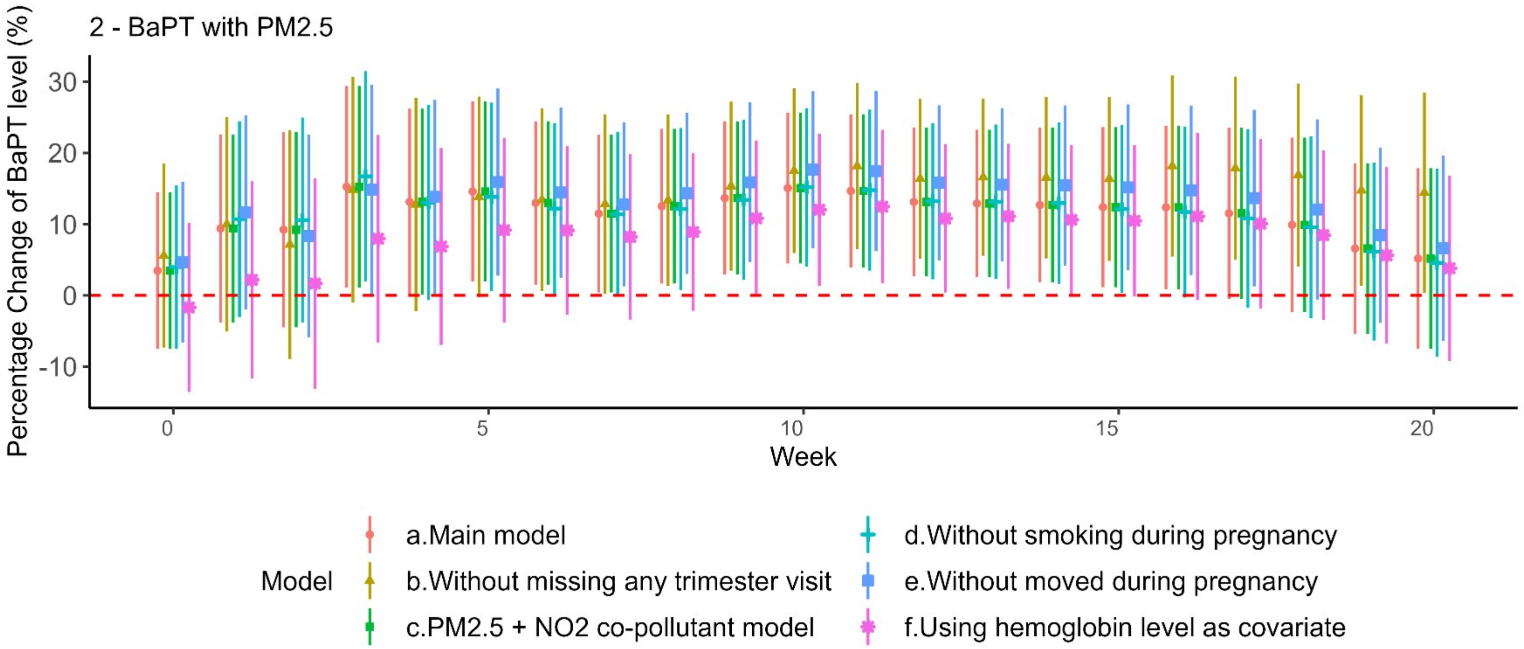

Figure S2. **Sensitive analysis: Difference of 1-Phenanthrene-Tetrols (PHET) and 2-Benzo(a)Pyrene-Tetrols (BaPT) concentrations association with one interquartile increase in Weekly cumulative $PM_{2.5}$**: a. main model, b. excluded participants who were missing one or two urine collections, c. included a co-pollutant $PM_{2.5}$ + NO_2 as covariates in the model, d. excluded participants who were smoking during pregnancy, e. excluded participants who moved to another address during pregnancy, f. using hemoglobin level as a covariate instead of normalization. Generated using mixed-effects models with random intercepts of study participants and controlling for age, gestation age, pre-pregnancy body mass index (BMI), income groups, education groups, and season.

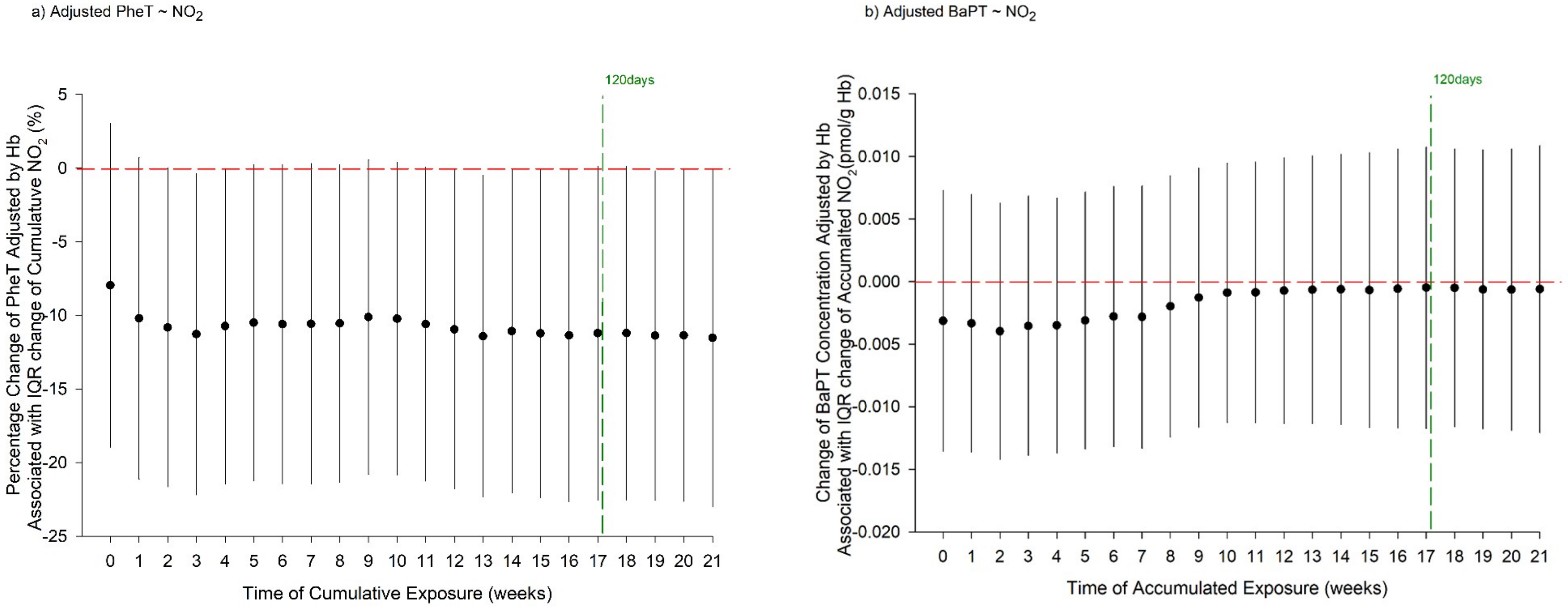

Figure S3. The Change of PAH-Tetrols Concentration with an IQR Change of Weekly Cumulative NO_2 Exposure: (a) Phenanthrene-Tetrols (PHET) Adjusted by Hemoglobin Levels; (b) Benzo(a)Pyrene-Tetrols (BaPT) Adjusted by Hemoglobin Levels. Generated using mixed-effects models with random intercepts of study participants and controlling for age, gestation age, pre-pregnancy body mass index (BMI), income groups, education groups.

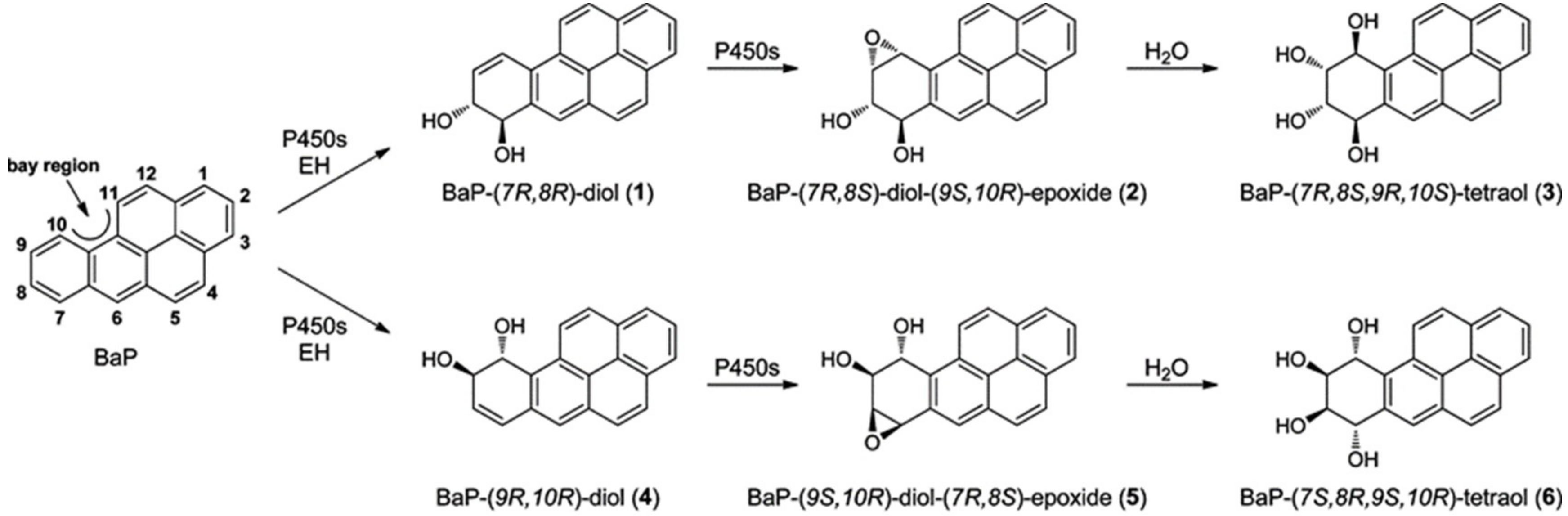

Figure S4. Metabolic Activation of Benzo(a)pyrene: Formation of Diols, Di-Epoxides, and Tetrols.

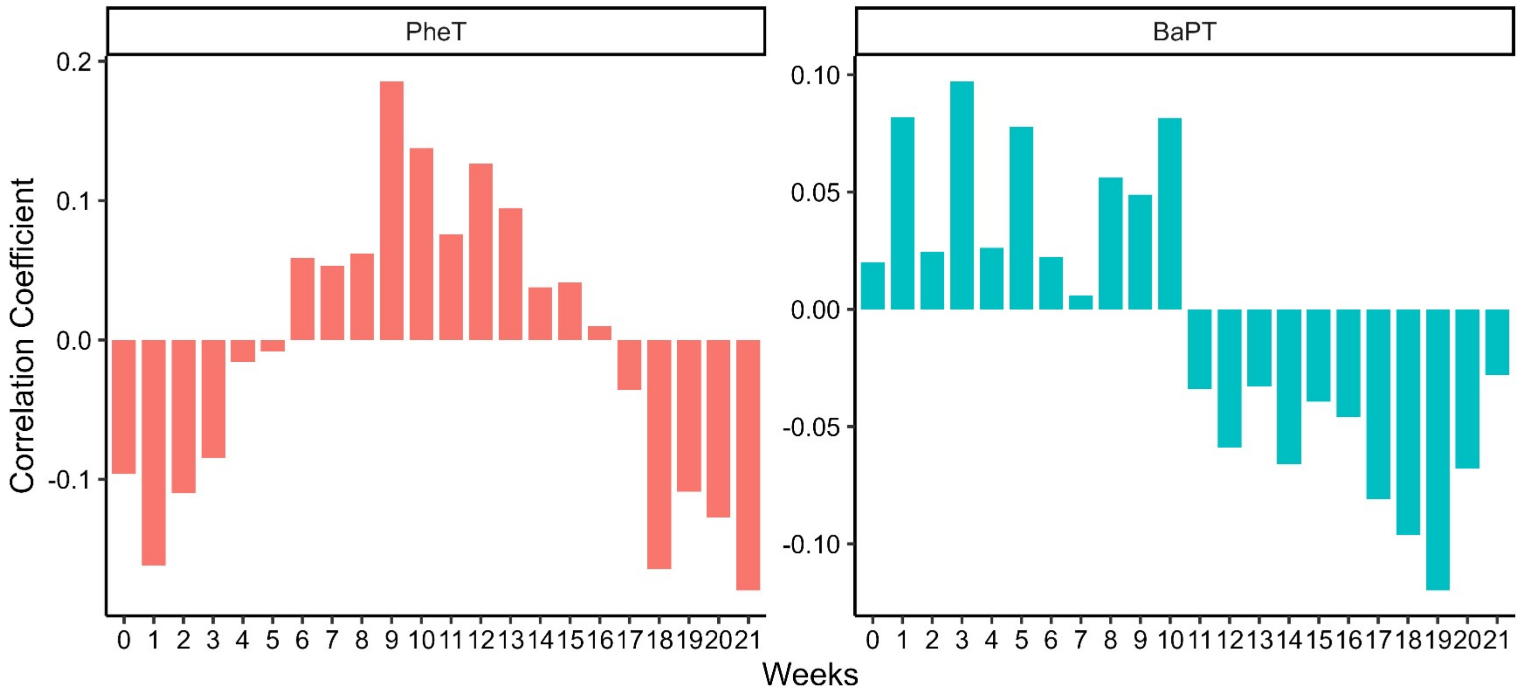

Figure S5. Pearson correlation coefficient between PAH tetrols with weekly cumulative PM2.5 concentration. PheT- Phenanthrene-Tetrols; BaPT- Benzo(a)Pyrene-Tetrols

Table S1-1. HPLC mobile phase gradient program

Time(min)	A%	B%	flow rate (μl/min)
0	90	10	400
2	90	10	400
4	40	60	400
6	40	60	400
12	20	80	400
14	0	100	400
16	0	100	400
18	40	60	400
20	90	10	400
22	90	10	400

Mobile phase : A: 0.01% formic acid in water; B: 0.01% formic acid in methanol

Table S1-2. Parameters used for the HPLC-MS/MS analysis

	PHET	BaPT
Ion Source Type	APCI	APCI
Polarity	Positive	Positive
Vaporizer Temperature	300	400
Sheath Gas Pressure	20	30
Aux Gas Pressure	5	5
Capillary Temperature	200	200
Capillary Offset	35	35

Table S2. Estimated percentage change of PAH-tetrols concentration associated with each interquartile range (IQR) change in cumulative $PM_{2.5}$ weekly concentrations.

1) **PheT ~ PM2.5**

Weeks	Percentage Change	95% CI Lower	95% CI Upper	p-value	IQR(μg/m³)
0	-2.36	-11.52	6.65	0.603	2.821
1	-8.27	-19.20	2.66	0.139	2.543
2	-9.75	-20.97	1.62	0.092	2.460
3	-8.71	-20.24	2.95	0.145	2.398
4	-3.84	-14.62	6.94	0.486	2.201
5	-1.92	-11.96	8.27	0.718	2.120
6	0.74	-8.42	9.90	0.874	1.906
7	2.22	-6.65	10.93	0.626	1.848
8	3.25	-5.47	11.82	0.465	1.773
9	5.76	-2.81	14.33	0.186	1.717
10	7.53	-1.03	16.10	0.084	1.644
11	8.27	-0.30	16.84	0.058	1.555
12	8.71	0.30	17.13	**0.042**	1.448
13	9.01	0.74	17.28	**0.033**	1.306
14	9.01	0.30	17.73	**0.042**	1.261
15	9.60	0.59	18.61	**0.036**	1.188
16	10.19	0.74	19.65	**0.035**	1.118
17	9.90	0.00	19.79	**0.050**	1.049
18	7.09	-3.25	17.28	0.180	0.972
19	5.02	-5.17	15.21	0.338	0.868
20	2.95	-7.98	13.88	0.591	0.855

2) **BaPT ~ PM2.5**

Weeks	Percentage Change	95% CI Lower	95% CI Upper	p-value	IQR(μg/m³)
0	3.48	-7.52	14.48	0.536	2.77
1	9.40	-3.81	22.61	0.164	2.52
2	9.23	-4.48	22.94	0.188	2.44
3	15.26	1.11	29.41	**0.035**	2.37
4	13.14	0.07	26.21	**0.049**	2.16
5	14.59	1.94	27.23	**0.024**	2.14
6	12.97	1.50	24.45	**0.027**	1.93
7	11.48	0.41	22.54	**0.043**	1.86
8	12.52	1.66	23.38	**0.024**	1.79
9	13.67	2.94	24.40	**0.013**	1.71
10	15.06	4.47	25.65	**0.006**	1.63

11	14.66	3.90	25.43	**0.008**	1.56
12	13.12	2.66	23.58	**0.014**	1.44
13	12.91	2.56	23.25	**0.015**	1.31
14	12.69	1.85	23.53	**0.022**	1.27
15	12.39	1.14	23.63	**0.031**	1.20
16	12.36	0.88	23.84	**0.035**	1.11
17	11.53	-0.47	23.53	0.060	1.05
18	9.90	-2.36	22.15	0.114	0.97
19	6.59	-5.38	18.55	0.281	0.86
20	5.17	-7.52	17.87	0.425	0.85

Table S3. Percentage changes of PAH-tetrols concentrations with one interquartile range (IQR) increase in proximity to a source traffic variable, with 95% confidence intervals and p-values.

Variable	Unit	Change (%)	CI Lower	CI Upper	P Value
**a. PHET**					
Distance to Airport	Km	1.13	-5.54	7.80	0.74
Distance to Railway Yard	Km	3.45	-0.82	7.73	0.11
AADT_300_RD	vehicles per year	1.07	-1.48	3.61	0.41
TruckAADT_300_RD	vehicles per year	1.10	-0.86	3.07	0.27
FastFoodWithin1000mBuffer	count	0.00	0.00	0.00	0.39
CrossingWithin1000mBuffer	count	0.41	-2.57	3.39	0.79
GasStationWithin1000mBuffer	count	0.00	0.00	0.00	0.27
RailwayWithin1000mBuffer	count	-0.89	-2.48	0.70	0.28
**b. BaPT**					
Distance to Airport	Km	-0.91	-1.88	0.07	0.07
Distance to Railway Yard	Km	-0.14	-0.77	0.48	0.65
AADT_300_RD	vehicles per year	0.24	-0.13	0.61	0.20
TruckAADT_300_RD	vehicles per year	0.17	-0.12	0.46	0.24
FastFoodWithin1000mBuffer	count	0.00	0.00	0.00	0.24
CrossingWithin1000mBuffer	count	0.24	-0.19	0.67	0.28
GasStationWithin1000mBuffer	count	0.00	0.00	0.00	0.42
RailwayWithin1000mBuffer	count	-0.22	-0.45	0.00	0.06

All models were adjusted for maternal age, gestation age, pre-pregnancy body mass index(BMI), income groups, education groups, sample collected season.

Abbreviations: AADT: annual average daily traffic.
